# Whole Genome Diversity, Population Structure, and Linkage Disequilibrium Analysis of Chickpea  (*Cicer arietinum* L.) Genotypes Using Genome-Wide DArTseq-Based SNP Markers

**DOI:** 10.3390/genes10090676

**Published:** 2019-09-04

**Authors:** Somayeh Farahani, Mojdeh Maleki, Rahim Mehrabi, Homayoun Kanouni, Armin Scheben, Jacqueline Batley, Reza Talebi

**Affiliations:** 1 Department of Plant Protection, Varamin-Pishva Branch, Islamic Azad University, Varamin P.O. Box 33817-74895, Iran; 2 Department of Biotechnology, College of Agriculture, Isfahan University of Technology, Isfahan P.O. Box 8415683111, Iran; 3 Kordestan Agricultural and Natural Resources and Education Center, Agricultural Research, Education and Extension Organization (AREEO), Sanandaj P.O. Box 714, Iran; 4 School of Biological Sciences, The University of Western Australia, Crawley, WA 6009, Australia; 5 Department of Agronomy & Plant Breeding, College of Agriculture, Sanandaj Branch, Islamic Azad University, Sanandaj P.O. Box 618, Iran

**Keywords:** chickpea, genetic diversity, linkage disequilibrium, DArTseq-SNP markers

## Abstract

Characterization of genetic diversity, population structure, and linkage disequilibrium is a prerequisite for proper management of breeding programs and conservation of genetic resources. In this study, 186 chickpea genotypes, including advanced “*Kabuli*” breeding lines and Iranian landrace “*Desi*” chickpea genotypes, were genotyped using DArTseq-Based single nucleotide polymorphism (SNP) markers. Out of 3339 SNPs, 1152 markers with known chromosomal position were selected for genome diversity analysis. The number of mapped SNP markers varied from 52 (LG8) to 378 (LG4), with an average of 144 SNPs per linkage group. The chromosome size that was covered by SNPs varied from 16,236.36 kbp (LG8) to 67,923.99 kbp (LG5), while LG4 showed a higher number of SNPs, with an average of 6.56 SNPs per Mbp. Polymorphism information content (PIC) value of SNP markers ranged from 0.05 to 0.50, with an average of 0.32, while the markers on LG4, LG6, and LG8 showed higher mean PIC value than average. Unweighted neighbor joining cluster analysis and Bayesian-based model population structure grouped chickpea genotypes into four distinct clusters. Principal component analysis (PCoA) and discriminant analysis of principal component (DAPC) results were consistent with that of the cluster and population structure analysis. Linkage disequilibrium (LD) was extensive and LD decay in chickpea germplasm was relatively low. A few markers showed r^2^ ≥ 0.8, while 2961 pairs of markers showed complete LD (r^2^ = 1), and a huge LD block was observed on LG4. High genetic diversity and low kinship value between pairs of genotypes suggest the presence of a high genetic diversity among the studied chickpea genotypes. This study also demonstrates the efficiency of DArTseq-based SNP genotyping for large-scale genome analysis in chickpea. The genotypic markers provided in this study are useful for various association mapping studies when combined with phenotypic data of different traits, such as seed yield, abiotic, and biotic stresses, and therefore can be efficiently used in breeding programs to improve chickpea.

## 1. Introduction

Chickpea (*Cicer arietinum* L.) is an important legume food crop that is currently cultivated in wide ranges of climatic regions across the world in more than 45 countries [1]. It is the second largest cultivated legume globally as a rich and cheap source of vegetarian protein, which plays an important role in human feed and nutritional security in most low income agricultural-based communities, such as Asia and Africa [2]. Chickpea is a self-pollinated diploid (2n = 2x = 16) plant with an approximate genome size of 931 Mbp [3] and comprises two types, *Desi* and *Kabuli* cultivars, that are distinctly different in agro-morphological characteristics such as seed shape, flower color, growth habit, and genome composition [2,4,5]. Both types of chickpea genotypes are grown worldwide, but *Desi* type is mainly cultivated in Ethiopia and Indian subcontinent [3]. The average world yield of chickpea is much lower than its potential yield under favorite conditions, mainly due to the narrow genetic base of improved cultivars and their vulnerability to various biotic and abiotic stresses [6,7]. Therefore, characterization of diverse germplasm is a fundamental prerequisite for plant breeders to select proper parental lines and utilize them in breeding programs. Classical breeding techniques based on morphological traits able to characterize genotypes based on their phenotypic characters, but these markers are limited in number, influenced by environment, and often have epistatic interaction with other traits. Molecular markers reflect the genetic diversity at the DNA level and are able to visualize the accurate genetic diversity between genotypes [8]. In chickpea, different DNA markers such as random amplified polymorphism DNA (RAPD) [9,10,11], inter-simple sequence repeat (ISSR) [12,13], amplified fragment length polymorphism (AFLP) [14,15], and simple sequence repeats (SSR) [16,17] have been used for genetic diversity analysis in different germplasm.

During the last decade, single nucleotide polymorphism (SNP) markers have been developed and increasingly utilized as highly-preferred molecular markers in various crop species because of their wide genome coverage, co-dominant inheritance, chromosome-specific location, low cost, and fast tracking in comparison to other polymerase chain reaction (PCR)-based molecular markers [18,19]. SNP markers are mainly developed based on next generation sequencing technology. Fast development of SNP markers through genotyping-by-sequencing (GBS) has paved the road to facilitating genomics-assisted breeding through quantitative trait loci (QTL) and genome-wide association analysis in diverse crops [20,21]. Recently, diversity array technology (DArT) developed a GBS method called “DArTseq” for genotyping with high-density SNP in different crop species such as wheat [22], common bean [23], sesame [8], tomato [24], snake melon [25], and chickpea [26].

In this study, we used DArTseq-based SNP markers for genetic diversity, population structure, and linkage disequilibrium analysis in 186 chickpea genotypes comprised of advanced *Kabuli* breeding lines and landrace *Desi* accessions.

## 2. Materials and Methods

### 2.1. Plant Materials and DNA Extraction

The 186 chickpea genotypes (Supplementary Table S1), including 20 Iranian landrace *Desi* accessions and 166 *Kabuli* advanced breeding lines supplied by the International Crops Research Institute for the Semi-Arid Tropics (ICRISAT) and the International Center for Agriculture Research in the Dry Areas (ICARDA), were employed for genetic diversity, linkage disequilibrium, and population structure analyses using DArTseq-based SNP markers. Fresh leaves of each genotype (pooled sample of ten plants per genotypes) were used for DNA extraction using the cetyltrimethyl ammonium bromide (CTAB) method [27], with minor modification (Supplementary Table S1). DNA concentrations were measured using spectrophotometer (Shimadzu UV-1800, Kyoto, Japan) and adjusted to 50 ng/µL.

### 2.2. Genotyping by DArTseq Platform

All chickpea genotypes were genotyped using sequencing-based DArT genotyping platform (DArT Pty Ltd, Canberra, Australia). This method is based on methyl filtration and next-generation sequencing platforms, as described before [28,29]. Initially, we received 6678 SNPs, which were polymorphic across chickpea genotypes. First, the markers with unknown chromosome position were removed from the analysis. The data set was filtered for minor allele frequency (MAF) lower than 0.1 and also for missing data higher than 10%. Chickpea is a self-pollinated crop, therefore, SNP markers that showed heterozygosity of more than 5% were also removed from the analysis. Overall, 1152 SNPs remained for further analysis of genetic diversity, population structure, and linkage disequilibrium in studied chickpea genotypes (summarized in Table S2). Quality of SNP markers were determined by the parameters “reproducibility” and “call rate”, as described previously [30].

### 2.3. Data Analysis

Polymorphism information content (PIC) values for SNP markers were calculated using PowerMarker v.3.25 [31]. Genetic distance and kinship matrix between pairs of 186 chickpea genotypes were computed using the identity-by-state (IBS) method implemented in TASSEL v.5.2.37 [32]. Cluster analysis of chickpea genotypes based on neighbor joining (NJ) [33] were imputed in DARwin ver 5.0 software [34]. Principal component analysis (PCA) for genotypes was imputed in TASSEL v.5.2.37 and the first two components were used for scatter plot distribution in XLSTAT 2012 (Addinsof, New York, NY, USA; www.xlstat.com). Functional annotation and prediction of effects on SNP markers were calculated in SnpEff v 4.2 [35]. Predicted impact of SNPs was categorized as low (synonymous substitution), moderate (non-synonymous substitution), high (disruptive impact on the protein), and modifier (with impact on noncoding regions).

In order to find the fitting grouping of chickpea genotypes, the genetic structure based on the Bayesian clustering approach was implemented by STRUCTURE 2.1 based on an admixture model [36], as in model the K-values ranging from 2 to 10 with 5 independent runs and burn-in period set at 100,000 and Markov Chain Monte Carlo (MCMC) repetitions after burn-in set at 100,000. The results of structure analyzed for estimate the optimal value of K using the Delta (K) method [36] were extracted using STRUCTURE HARVESTER web version 0.6.94 [37]. Genetic diversity among and between populations, proportion of chickpea genotype membership in each cluster, and Wright’s F-statistics (FST) among subpopulations were extracted from STRUCTURE 2.1.

In order to finding the fitting pupation structure, we complemented the STRUCTURE analysis with the discriminant analysis of principal components (DAPC) analysis using the procedure proposed by the R’s package “adegenet” [38,39]. Linkage disequilibrium (LD) for SNP markers was implemented in TASSEL v.5.2.37 and graphical LD decay was imputed by GAPIT R package [40].

## 3. Results

### 3.1. Quality, Diversity, and Functional Characterization of SNPs

**Figure 1:**
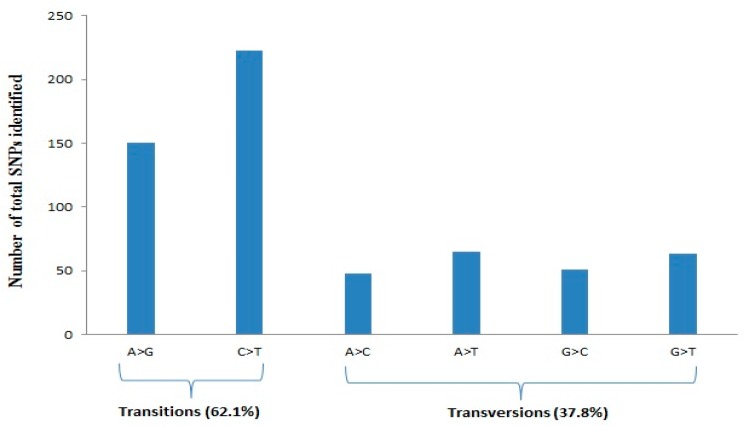
Percentage distribution of transition and transversion SNPs identified using DArTseq assay.

The 186 chickpea genotypes were analyzed by DArTseq-SNPs. The initial data set consisted of 3339 SNPs and after filtering data for some quality parameters, including minor allele frequency lower than 0.1, missing data ≥10%, and also SNPs with unknown chromosome position, a total of 1152 SNPs were selected for genome diversity analysis (Supplementary Table S2). The number of mapped SNP markers varied from 52 (LG8) to 378 (LG4), with an average of 144 SNPs per linkage group (Table 1). As shown in Table 1, the chromosome size covered by SNPs varied from 16,236.36 kbp (LG8) to 67,923.99 kbp (LG5), while LG4 showed a higher number of SNPs, with an average of 6.56 SNPs per Mbp (Table 1). Quality parameters such as call rate and average reproducibility were 0.97 and 0.98, respectively. PIC values of SNP markers ranged from 0.05 to 0.50, with an average of 0.32, while the markers on LG4, LG6, and LG8 showed higher mean PIC values than the average (Table 1). A higher frequency of A/G and C/T transitions (62.1%) was evident compared to A/C and G/T transversions (Figure 1).

**Figure 2:**
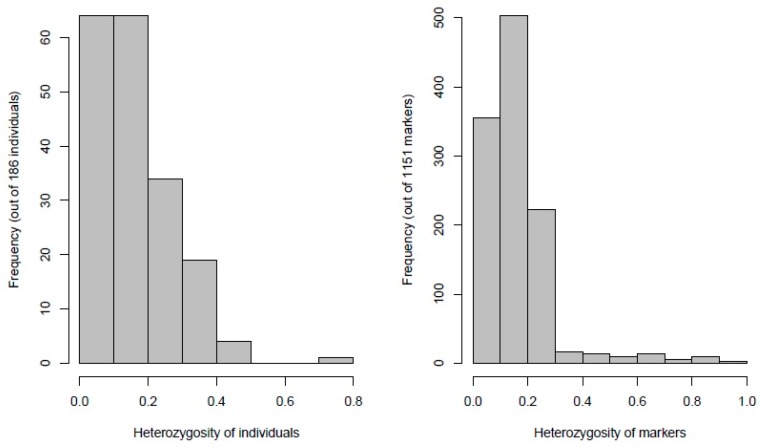
Frequency of heterozygous genotypes and heterozygosity of 1152 SNP markers generated by DArTseq platform in 186 chickpea genotypes.

Distribution of heterozygous chickpea genotypes and SNP markers revealed low values of heterozygosity (>0.2) in more than 75% of chickpea genotypes and SNP markers (Figure 2). These observations are to be expected since chickpea is a highly self-pollinated crop. Functional annotation of 1152 SNP variants showed the total of SNPs in genes was 917 (79.6%), of which (545) 59.4% were in exons. A total of 6669 functional effects for SNP variants were predicted for 1152 SNPs. The predicted effects were of modifier type (88.38%), moderate impact (3.72%), low impact (7.38%), and high impact (0.53%).

### 3.2. Genetic Distance and Relatedness Between Chickpea Genotypes

**Figure 3:**
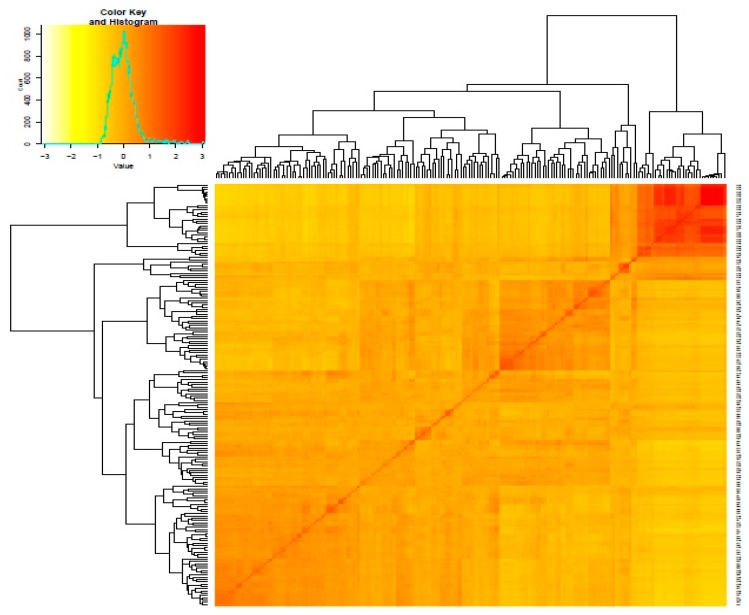
Heat map plot of kinship matrix using average linkage clustering based on SNP markers depicts the existence of four different groups among chickpea genotypes. The details of members of these groups are presented in Table S3.

**Figure 4:**
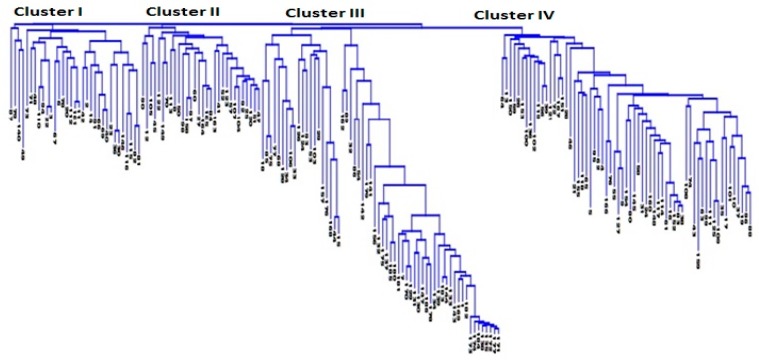
The neighbor joining cluster analysis using DArTseq-SNP markers for grouping 186 chickpea genotypes.

Kinship coefficient between pairs of chickpea genotypes varied from −0.83 to 3 (on a scale of −3 to 3). Overall, 66% of the pairs of 186 chickpea genotypes had kinship values of ≤0.5 (Supplementary Table S3). Kinship matrix obtained from DArTseq-SNP markers resulted in four distinct groups (Figure 3). In order to identify the most similar pairs of genotypes, genetic distance matrix was computed between pairs of genotypes, which varied from 0.02 to 0.56, with an average of 0.31 (Supplementary Table S4). The large proportion (88%) of pairs of genotypes showed genetic distance ≥0.25 (Supplementary Table S4). The Neighbor Joining (NJ) cluster analysis based on DArTseq-SNP markers differentiated chickpea genotypes into four clusters (Figure 4). Cluster I comprised 33 genotypes, all of which are advanced breeding lines originated from ICARDA. Most of the genotypes in this cluster have the same parents (X2002TH or FLIP98-38C) in their pedigrees. Cluster III contained Iranian *Desi* landraces and two Iranian improved *Desi* cultivar (Kaka and Pirouz). Clusters III and IV were larger groups of chickpea genotypes compared to cluster I and II. Most of the genotypes that originated from ICRISAT grouped in cluster IV. There were no strong relationships between cluster grouping and pedigree of chickpea genotypes, although most of *Desi* chickpea landraces grouped in cluster III, and in cluster II and cluster IV most of the genotypes showed at least one or two of the same parents in their pedigrees. Most of the genotypes used in this study have been used as parental lines or have similar genetic backgrounds, so a mixture of pedigree is observed in all clusters.

### 3.3. Population Structure and Discriminate Analysis of Principal Coordinate (DAPC)

**Figure 5:**
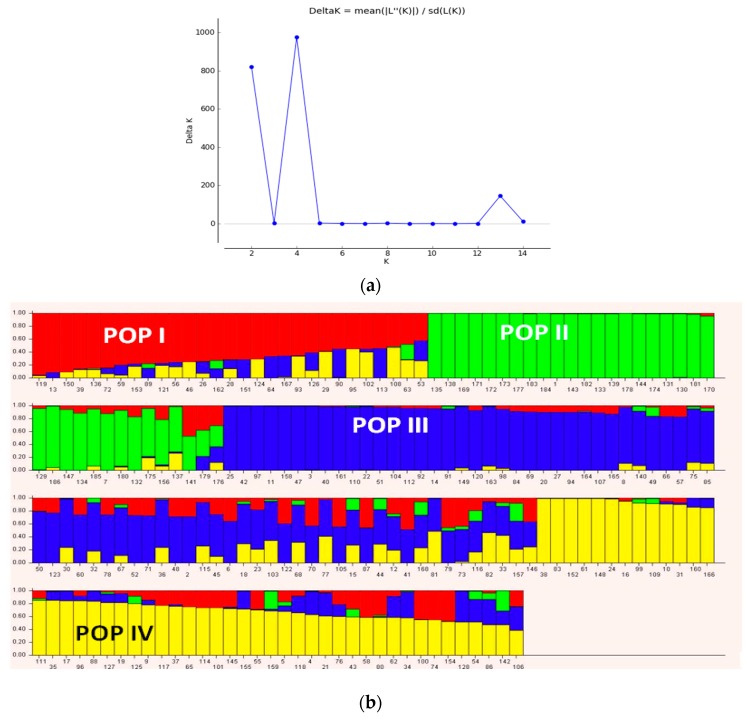
Determination of the optimal value of *K* = 4 and population structure of 186 chickpea genotypes using DArTseq-SNP markers.

Population structure analysis of the genotypes based on the Bayesian model implemented in STRUCTURE software grouped genotypes at K = 4 (Figure 5). Four sub-populations based on SNP markers showed relatively low genetic divergence among sub-populations (from 0.19 for POP4 to 0.28 for POP1), while high divergence between sub-populations was observed (Table 2). Genetic diversity among the populations based on net nucleotide distance revealed a higher distance between POP1 and POP2 compared to the genetic distance between POP1 and POP2 with POP3 and POP4 (Table 2). Mean fixation index of sub-populations ranged from 0.56 (POP1) to 0.65 (POP3) (Table 2). Admixed of ancestry was not detected in 21.5% (40 genotypes) of chickpea genotypes, whereas the remaining genotypes showed 5–40% admixed ancestry (Supplementary Table S1). The highest admixed ancestry was observed between POP3 with POP1 and POP4, compared to that obtained between POP2 and POP3. This could be associated to the same parents (FLIP98-28C, FLIP98-38C and FLIP98-52C) that were used in pedigree of the most advanced breeding lines from ICARDA. Admixed genotypes in cluster III contains all landrace *Desi* accessions that collected from North West of Iran.

**Figure 6:**
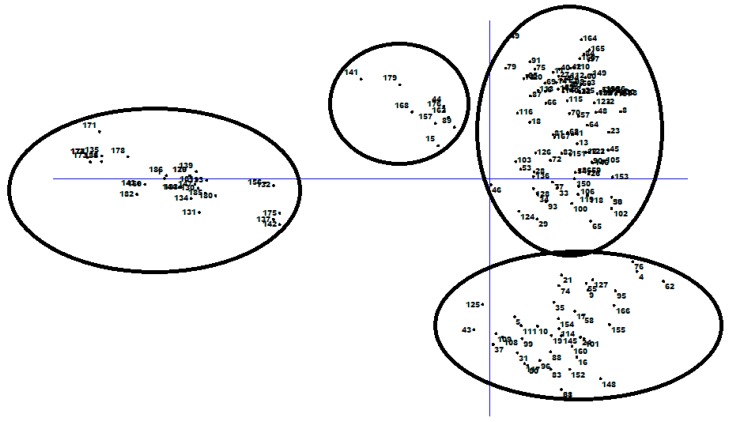
Principal coordinate analysis of 186 chickpea genotypes based on DArTseq-SNP markers.

**Figure 7:**
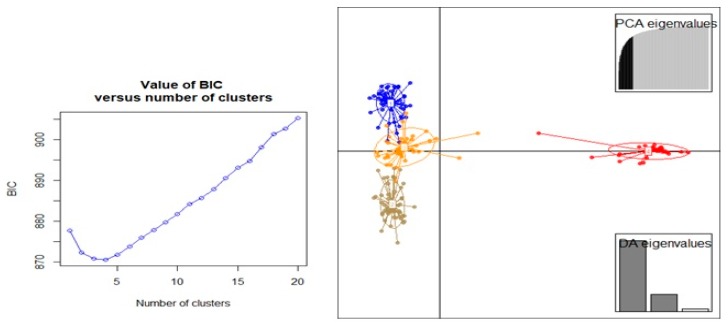
The percentage of cumulative variance for the retained PCA eigen vectors and scatter plot from the DAPC analysis for 186 chickpea genotypes used to determine the optimal *k* number of clusters using DArTseq-SNP markers.

Principal component analysis (PCA) based on DArTseq-SNP markers revealed four distinct groups of chickpea genotypes and two principal components, accounting for 75.18% of total variation (Figure 6). Discriminant analysis of principal component (DAPC) also was employed to find the fitting population structure based on DArTseq-SNP markers. The lowest Bayesian information criterion (BIC) value was obtained at K = 4, therefore, three discrimination functions were detected, which explains 30.17, 25.86, and 19.06% of the variation between sub-groups (Figure 7). Results from the DAPC analysis were consistent with the results from the population structure analysis.

### 3.4. Linkage Disequilibrium Analysis

**Figure 8:**
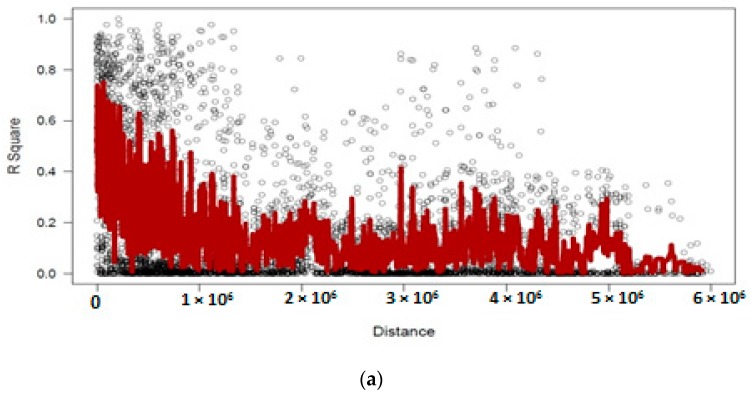

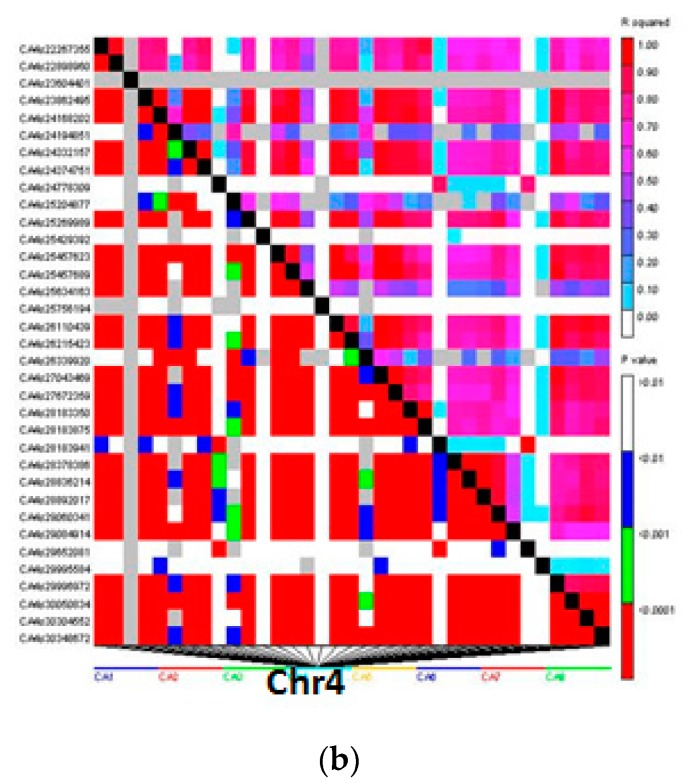
Linkage disequilibrium (LD)-measured *r*^2^ plotted vs. the physical map (bp) between pairs of DArTseq-SNP markers in a panel of 186 chickpea genotypes (**a**), which show the huge LD decay on Chr4 (**b**).

Distribution of LD within chromosomes based on 1152 DArTseq-SNP markers showed extensive LD decay, as in the entire population from 56,325 marker pairs, 21,660 (38.4%) intra-chromosomal pairs showed a significant level (*p* < 0.001) of LD. Mean r^2^ value was 0.22, while the critical r^2^ value was 0.33. The overall LD decay in chickpea germplasm was relatively low and few markers showed r^2^ ≥ 0.8. Nevertheless 2961 pairs of markers showed complete LD (r^2^ = 1), although a huge LD block was observed on LG4 (Figure 8).

## 4. Discussion

Characterization of genetic diversity in crop species is a prerequisite for efficient conservation and utilization of germplasm and developing breeding programs [10,41]. Iran, Afghanistan, Turkey, Indian subcontinent, and Lebanon contain a large number of chickpea landraces and have been previously identified as the centers of origin and/or diversity of chickpea by Vavilov [42]. Most chickpea growing areas worldwide are under biotic and abiotic stresses, resulting in low seed yield production. This is mainly due to the narrow genetic base and lack of desirable traits in cultivated genotypes [42,43]. Therefore, incorporation of desirable traits with a high rate of allelic frequencies and transgressive segregation through introduction of diverse genotypes from diverse sources into breeding programs is required to improve tolerance to various stresses and to maximize seed yield and quality [26,44]. To date, different molecular markers have been utilized for genetic diversity analysis in chickpea [11,12,13,14,17]. Marker-assisted breeding in chickpea is usually hindered due to low genetic diversity and low intra-specific polymorphism among *Desi* and *Kabuli* chickpea geneotypes. Therefore, development and implementation of large-scale informative markers like SNPs assist breeders in differentiating the chickpea germplasm at a genome-wide scale [21]. DArTseq-SNP markers conducted by GBS technology is a rapid, low cost, and efficient method for genotyping, providing a broad genome coverage, and therefore has been increasingly were used in different plants species [23,24,25], as well as in chickpea [26]. In general, the genetic diversity estimated by SNPs may be lower than those estimated through SSR markers; however, the accurate consideration of genetic diversity reflected to the number of loci instead of the number of alleles [8]. Therefore, sufficiently large numbers of next-generation-based SNPs are analyzed across the genome and are able to estimate accurate genome-wide diversity in different crop species.

In this study, we employed DArTseq-SNP markers for population structure and LD analysis in a set of 186 diverse chickpea genotypes. The generally low sequence diversity in self-pollinated crops like chickpea may increase the correlation among markers [8,16,19,26]. Therefore, for precision diversity and association analysis, a reduced set of markers is needed. Thus, we filtered the 3339 SNPs and 1152 markers (34.5%) for further analysis. The average call rate and reproducibility of SNP markers was 0.97 and 0.98, respectively, which was higher than values previously reported in watermelon [45] and common bean [23], and was consistent with the value reported for wheat [46,47]. The average PIC value of SNP markers was 0.32, and more than half of the markers showed PIC values higher than 0.25, which suggests sufficient efficiency of these markers that have been reported previously for SNP markers in chickpea [19,26,48]. Physical distribution of the mapped markers with known positions on chickpea linkage groups (Table 1) show that LG4 had a higher marker density compared to other LGs, which is consistent with previous studies [19,26].

The structural and functional annotation of 1152 SNPs show a high number of SNPs (917) in gene regions, of which 545 (47.3%) were in exons. This estimated ratio using DArTseq-based SNP markers in chickpea genes is comparable with previously reported orthologous chickpea genes/transcripts [2,4]. Therefore, these informative DArTseq-based SNPs identified in genic regions can be utilized as powerful markers for genome-wide association mapping or QTL analysis for identifying major candidate genes associated with resistance to biotic and abiotic stresses in chickpea [19].

Chickpea is a self-pollinated crop, and it is assumed that all chickpea genotypes in the present study had been held several generations via self-pollination; therefore, they were expected to be mostly homozygous. The low level of heterozygosity in the chickpea panel suggests that the genotypes we used in this study were close to being inbred lines and homozygous. Average inter-chromosomal LD decay of SNP markers showed long distances for marker pairs in LD (Figure 8), which may be attributed to genetic admixture, apart from the genetics or physical distances as has been previously reported in chickpea and other crops [26,49].

Kinship values calculated between pairs of genotypes are a reliable factor for understanding the extent of relatedness between chickpea genotypes [24,50]. Kinship values close to zero indicate unrelated germplasm, while those close to 0.5 or higher (which around 66% of the pairs of genotypes in this study had) refer to full sibs or highly similar germplasm. Average genetic distance (GD) between pairs of genotypes was 0.31, while 88% of pairs of genotypes showed GD of more than 0.25, indicating that high genetic variability were presented among chickpea genotypes. The different approaches (STRUCTURE, PCoA, and DAPC) used to analyze the structure of the chickpea germplasm appeared to provide complementary information. The neighbor joining tree divided the chickpea genotypes into four main clusters, which are in complete concordance with the structure and PCoA analysis results. These results suggest that the crossing among inter-cluster genotypes may develop cultivars with promising agronomic traits.

The use of the cluster analysis and population structure was in concordance with those of DAPC and showed better performance and allowed for a population subdivision. However, some groups were composed of a mixture of chickpea genotypes, and some genotypes were included in different clusters, which could be due mainly to the same parental lines that were used in the breeding pedigree of these genotypes.

This might be due to the fact that these genotypes were utilized in different breeding programs, and thus they have same parental pedigree, as shown in Table S1 that FLIP98-28C and FLIP98-52C included in pedigree of large number of chickpea genotypes. Therefore, it is more likely that recent breeding activities and incorporation of the same genotypes in parental crossing, as well as domestication and selection of similar chickpea genotypes by farmers during past centuries, had a significant impact on global chickpea genetic structure, resulting in genotypic admixture as shown in this study and previous reports [51,52]. Therefore, breeding activities with different strategies and that incorporate the same genotypes in parental crossing may led to significant impact on global chickpea genetic structure [53]. The largest chickpea germplasm collection maintained at the International Center for Agricultural Research in the Dry Areas (ICARDA), International Crops Research Institute for the Semi-Arid Tropics (ICRISAT), and in chickpea originated centers like Iran. These genotypes have served—and continue to serve—as sources of genes for desirable traits contributing to the successes being recorded in variety development, especially for resistance to environmental stresses [17,20]. Interestingly, many of the genotypes used in this research were studied for the first time and have not been previously used in breeding programs. Therefore, these genotypes are appropriate novel sources that may possess useful genes and can be used in breeding programs to broaden the chickpea gene pool.

## 5. Conclusions

In this study, we employed DArTseq-SNP markers for genome diversity, population structure, and LD analyses in a mini-core collection of advanced chickpea breeding lines and landraces. The large number and high genome coverage of these markers showed high polymorphism in the studied chickpea germplasm, which is a valuable and remarkable tool for genome-wide screening of genetic diversity in chickpea. This genetic diversity of the chickpea gene pool will be highly valuable information for various purposes, including allele mining and donor parent selection for developing new improved chickpea germplasm for target traits of interest. The appropriate DArTseq-SNP markers for different gene/traits of interest can be used for cloning and designing competitive allele specific PCR (KASP) in chickpea.

Therefore, our next objective is to identify chickpea genotypes with desirable traits, and to conduct association mapping studies focusing on resistance to biotic stresses (*Ascochyta rabiei* and *Fusarium oxysporum*) and seed yield under environmental stresses like drought.

## Figures and Tables

**Table 1:** Polymorphism information content (PIC), call rate, average reproducibility, and distribution of DArTseq-SNPs on chickpea chromosomes.

Linkage Group (Chromosome)	Number of SNPs	Chromosome Size (kbp)	Mean of SNPs per Mbp	PIC Range (Mean)	Call Rate	Average Reproducibility
LG1	192	44,634.56	4.20	0.05–0.49 (0.23)	0.96	0.98
LG2	89	36,915.99	2.41	0.05–0.49 (0.32)	0.96	0.98
LG3	105	61,351.17	1.71	0.05–0.49 (0.30)	0.97	0.98
LG4	378	57,562.47	6.56	0.05–0.50 (0.36)	0.96	0.97
LG5	74	67,923.99	1.08	0.09–0.48 (0.31)	0.97	0.98
LG6	141	63,087.8	2.34	0.05–0.50 (0.34)	0.97	0.97
LG7	121	54,252.93	2.23	0.05–0.50 (0.31)	0.97	0.98
LG8	52	16,236.36	3.20	0.06–0.49 (0.34)	0.97	0.97
Total	1152	67,923.99	16.96	0.05–0.50 (0.32)	0.97	0.98

**Table 2:** Genetic divergence among (net nucleotide distance) and within (expected heterozygosity) population, proportion of membership, and mean value of Fst observed from the study of the population structure of 186 chickpea genotypes using DArTseq-SNP markers.

Population	Net Nucleotide Distance			Expected Heterozygosity	% of Membership	Mean Fixation Index (Fst)
	POP2	POP3	POP4			
POP1	0.44	0.25	0.26	0.28	0.20	0.56
POP2		0.33	0.30	0.22	0.19	0.62
POP3			0.16	0.17	0.36	0.65
POP4				0.19	0.26	0.63

## Supplementary Materials

The following are available online at https://www.mdpi.com/2073-4425/10/9/676/s1, Table S1: List and pedigree of 186 chickpea genotypes used for DArTseq-SNP genotyping; Table S2: List of DArTseq-SNP markers used in this study; Table S3: Kinship matrix between pairs of 186 chickpea genotypes based on DArTseq-SNP markers; Table S4: Genetic distance matrix of 186 chickpea genotypes based on DArTseq-SNP markers.
